# An Interpretable Soft-Sensor Framework for Dissertation Peer Review Using BERT

**DOI:** 10.3390/s25206411

**Published:** 2025-10-17

**Authors:** Meng Wang, Jincheng Su, Zhide Chen, Wencheng Yang, Xu Yang

**Affiliations:** 1 Graduate School & School of Computer and Cyberspace Security, Fujian Normal University, Fuzhou 350108, China; wangmeng@fjnu.edu.cn (M.W.); qsx20231393@student.fjnu.edu.cn (J.S.); 2 School of Mathematics, Physics and Computing, University of Southern Queensland, Queensland 4350, Australia; wencheng.yang@unisq.edu.au; 3 School of Computer and Data Science, Minjiang University, Fuzhou 350108, China; xu.yang@mju.edu.cn

**Keywords:** dissertation reviews, BERT-based model, Shapley Additive exPlanations(SHAP), interpretable soft-sensor

## Abstract

Graduate education has entered the era of big data, and systematic analysis of dissertation evaluations has become crucial for quality monitoring. However, the complexity and subjectivity inherent in peer-review texts pose significant challenges for automated analysis. While natural language processing (NLP) offers potential solutions, most existing methods fail to adequately capture nuanced disciplinary criteria or provide interpretable inferences for educators. Inspired by soft-sensor, this study employs a BERT-based model enhanced with additional attention mechanisms to quantify latent evaluation dimensions from dissertation reviews. The framework integrates Shapley Additive exPlanations (SHAP) to ensure the interpretability of model predictions, combining deep semantic modeling with SHAP to quantify characteristic importance in academic evaluation. The experimental results demonstrate that the implemented model outperforms baseline methods in accuracy, precision, recall, and F1-score. Furthermore, its interpretability mechanism reveals key evaluation dimensions experts prioritize during the paper assessment. This analytical framework establishes an interpretable soft-sensor paradigm that bridges NLP with substantive review principles, providing actionable insights for enhancing dissertation improvement strategies.

## 1. Introduction

Within the realm of academic research, peer reviews constitute an indispensable mechanism for safeguarding its integrity and advancement [1]. Not only do they uphold academic standards, but they also cultivate critical thinking and refine research through expert feedback, establishing themselves as a cornerstone of academic development [2]. This rigorous process involves subjecting the scholarly work to the scrutiny of domain experts to validate its reliability, validity, and originality. However, traditional peer-review methodologies—predominantly reliant on manual assessment and qualitative analysis—are becoming increasingly inadequate. These approaches are overwhelmed by the growing volume and complexity of scientific production, a challenge exacerbated by the demand for faster and broader dissemination [3]. Bauchner [4] pointed out that artificial intelligence should be used to assist peer-review publication.

Driven by rapid advances in natural language processing (NLP), particularly the emergence of deep learning models, novel avenues for enhancing peer-review methodologies have emerged [5]. Modern NLP facilitates the automated processing of large quantities of textual data, enables quantitative analysis, and uncovers complex underlying patterns [6]. For instance, by leveraging automated text classification and sentiment analysis, NLP can differentiate between constructive suggestions and critical commentary, as well as identify emotional nuances within reviewer feedback [7]. This capability holds significant practical value for graduate students in refining dissertation writing and effectively addressing reviewer critiques. Nevertheless, conventional NLP approaches often fail to capture the intricate semantic relationships and contextual dependencies inherent in review texts, resulting in superficial analyses that overlook latent evaluation patterns and substantive quality indicators embedded in expert feedback.

To overcome these limitations, this study draws inspiration from the concept of sensors, prevalent in industrial process control, to reframe automated review analysis for dissertations. The approach positions the soft-sensor as an essential supplement that extends data acquisition beyond physical hardware, targeting the wealth of data from software platforms. This academic soft-sensor aims to infer latent, difficult-to-quantify dimensions of scholarly quality from these digital streams. The proposed interpretable BERT-based soft-sensor framework thus plays a critical role in building a comprehensive data-driven educational system by transforming subjective textual feedback into quantifiable assessments. Furthermore, by integrating SHAP (Shapley Additive exPlanations), the framework provides a calibration and output interface, translating the model’s complex inferences into human-interpretable insights and thereby bridging the gap between black-box NLP models and actionable evaluative principles.

In summary, the key contributions of this study can be summarized as follows:

1. An enhanced BERT encoder architecture is proposed as the core inference engine of the soft-sensor. By integrating an additional self-attention layer, it significantly improves the ability to capture critical textual features, with experimental results demonstrating substantial gains in performance metrics compared to traditional baselines.
2. SHAP value analysis is introduced as an interpretability interface for the soft-sensor, enabling quantitative identification of key dimensions influencing evaluation outcomes in peer-reviewed texts. This approach offers novel insights into the reviewer decision-making process.
3. The soft-sensor framework is leveraged to systematically quantify factors prioritized by reviewers during assessment, providing actionable insights for manuscript improvement while supporting data-driven research into academic peer-review mechanisms.

The rest of our paper is organized as follows. Section 2 delves into the foundational concepts, including BERT, self-attention mechanisms, and SHAP. Section 3 critically reviews the related literature. Section 4 elaborates on the construction of the interpretable soft-sensor, detailing its implementation from data collection and preprocessing to text classification using BERT and significance analysis of words via SHAP. Section 5 evaluates our proposed framework through various metrics, performance comparisons, and detailed result analysis. Section 6 discusses the limitations of this work and future directions for research. Finally, Section 7 provides a comprehensive summary of the paper.

## 2. Background Concepts

### 2.1. BERT

**Figure 1:**
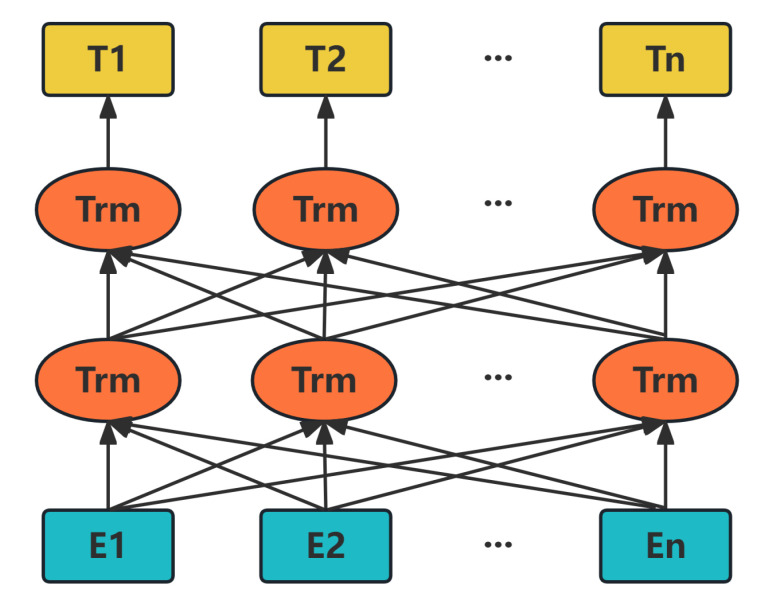
BERT network structure.

In natural language processing, pre-trained language models have become a key technology to improve the performance of various downstream tasks. BERT (Bidirectional Encoder Representations from Transformers) is a model proposed by Devlin [8], which mainly learns deep representations of language through large-scale unsupervised pre-training. The key feature of the model lies in its utilization of bidirectional Transformer encoders coupled with pre-training tasks, including Masked Language Model (MLM) and Next Sentence Prediction (NSP). The multi-head self-attention mechanism in each encoder layer and the feed-forward neural network work together to enable BERT to effectively integrate information at different locations, thus achieving advanced performance on multiple language understanding tasks such as question answering, natural language inference, and sentiment analysis [9,10]. This design makes BERT excellent at capturing the bidirectional structure of language, setting a new performance benchmark for natural language processing techniques. The structure diagram of the BERT model is shown in Figure 1—the input layer of the model consists of a series of yellow squares E1, E2, ..., EN which denote and represent the embedded representation of the input tokens. The Transformer (Trm) layers sequentially process the embedded inputs through multi-head self-attention and feed-forward operations. Each square of the output layer T1, T2, ..., TN represents the final encoded representation of the input token after being processed by all Transformer layers.

### 2.2. Self-Attention

**Figure 2:**
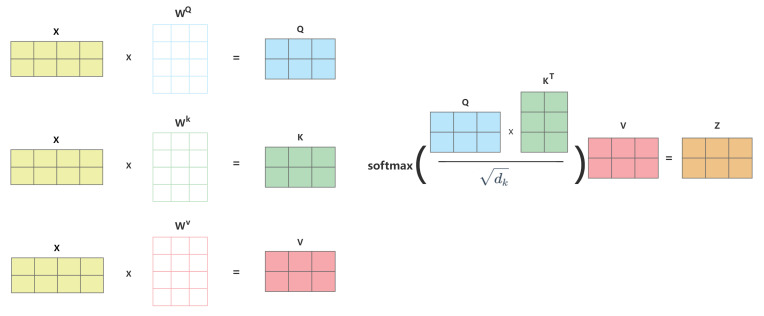
Self-attention mechanism diagram.

The self-attention mechanism is an effective method for sequence modeling and feature extraction. This mechanism was first widely recognized in the Transformer model proposed by Vaswani et al. [11]. This mechanism extracts features by calculating the association weight of each element to other elements in the sequence, allowing the model to capture long-distance dependencies and improving the efficiency of parallel computation. Compared with the traditional recurrent neural network, the self-attention structure is more efficient in processing long sequences and has become the basis of many deep learning architectures. As shown in Figure 2, in the self-attention computation, the input data *X* is first linearly transformed by the weight matrices $W_{q}$, $W_{k}$, $W_{v}$ to generate three new tensors: query, key, and value. Next, the query is multiplied by the transpose of the key matrix to compute the relevance scores among the words, followed by scaling the results to prevent enormous values. Subsequently, the softmax function is applied to normalize these relevance scores, yielding the normalized associations among words. Finally, the normalized relevance matrix is multiplied by the value matrix *V*, resulting in a weighted sum that produces new vector encodings for each word.

### 2.3. SHAP

SHAP (Shapley Additive exPlanations) is an advanced model explanation method based on game theory, aiming to improve machine learning models’ transparency and interpretability. This approach uses the notion of the Shapley value, a fair way of distributing payments in cooperative games introduced by Lloyd Shapley [12]. By calculating the average contribution of each feature in all possible feature combinations, SHAP applies not only to various machine learning models, including complex nonlinear models such as deep learning and ensemble methods but also to ensure fairness and consistency of interpretation [13]. In the SHAP value calculation, a machine learning model is assumed, *f*, whose input is the feature vector *x*, and output is the predicted value f(x). For the feature set *S*, the SHAP value $\phi_{i}$ is calculated as follows:

$$\phi_{i}=\sum_{S\subseteq N\setminus \{i\}}\frac{|S|!(|N|-|S|-1)!}{|N|!}\left(f(S\cup \{i\})-f(S)\right)$$

where *N* is the set of all features and $\left|S\right|$ is the features’ number of set *S*. This formulation takes into account the influence of features in different combinations and quantifies the specific contribution of each feature to the model prediction. The SHAP value of a feature reflects its specific contribution to the prediction result of the model, thus enabling researchers and practitioners to weigh the influence of each input feature systematically.

## 3. Related Work

### 3.1. Peer Review

Peer review is essential in graduate education as a critical mechanism of academic quality control. However, with the expansion of the scale of graduate education, the traditional paper review method faces the challenge of efficiency and objectivity. The academic community has shown strong interest in improving the peer-review process in recent years. Li [14] employed three tools—LIWC, SentimentR, and Stanford CoreNLP—to evaluate the emotional tones of peer-review comments. Additionally, Buljan et al. [15] utilized text analysis software to conduct a thorough analysis of 472,449 peer-review reports, examining multiple dimensions including tone, authenticity, impact, emotions, and ethics. With the advent of large language models (LLMs), Kousha et al. [16] conducted a systematic review and discussion on how LLMs can enable the partial or complete automation of tasks related to paper review processes. Meanwhile, Jin et al. [17] developed a peer-review simulation framework that leverages LLMs, effectively decoupling the influences of multiple potential factors and offering valuable insights for improving the design of peer-review mechanisms.

In the era of the rapid development of information technology, exploring the effective combination of AI technology and open science is an essential direction for the future development of peer review.

### 3.2. Applications of NLP

NLP technology has been widely used in text analysis, covering many aspects, from basic text processing to complex semantic analysis. With the development of machine learning models, especially the application of deep learning, the application of NLP technology in the field of higher education is getting deeper and deeper.

Research by Kastrati et al. [18] shows that sentiment analysis techniques have been widely used to analyze student feedback on learning platforms. However, the field is still growing rapidly in the face of informal and diverse student language and the processing challenges of large amounts of data, especially in applying deep learning. Further, Wu et al. [19]’s review, covering 2480 studies, generalizes the use of NLP in education into five domains by applying neural topic modeling and pre-trained language models, highlighting the shift from technology-centric to problem-oriented research topics, suggesting that NLP techniques are increasingly being tailored to specific challenges in education. In addition, in the academic peer-review process, the opposition-sentence attention (OSA) mechanism introduced by Lin et al. [20] significantly improved the prediction accuracy of the overall peer-review recommendations by prioritizing sentences with robust opposition-related learning through positive unlabeled learning methods.

These studies advance our understanding of educational feedback and interaction and drive innovation in educational research methods and applications, highlighting the potential value of NLP in educational progress.

With the development of pre-trained language models such as BERT, the application of NLP in text analysis has reached new heights, further enhancing the ability to analyze education-related texts and facilitating performance improvements on multiple tasks.

### 3.3. BERT in Text Classification

Text classification, a fundamental task in NLP, has undergone a transformative shift with the advent of pre-trained language models, particularly BERT. This revolutionary technique has emerged as a cornerstone for enhancing text classification performance, opening up new possibilities in the field.

BERT models, with their unique ability for fine-grained classification, find practical applications in tasks like sentiment analysis and intent recognition. The effectiveness of BERT models has been empirically validated in domains such as movie review analysis and consumer feedback sentiment analysis, as demonstrated by their performance on benchmark datasets like IMDb and Yelp [21,22]. Sun et al. [23] further validate this by showcasing BERT’s accuracy in capturing emotional changes and subtle tendencies in text.

Furthermore, compared with the traditional Word2Vec word embedding, BERT shows advantages in feature extraction, feature fusion, and model generalization. Lang Cong’s research [24] proves this through experiments. At the same time, Zheng et al. [25] combined BERT with deep learning to apply to the sentiment analysis of microblog text, which achieved better results compared with traditional deep learning methods.

BERT’s high performance is not limited to a single model application. When combined with networks such as BiLSTM and BiGRU, the hybrid model formed by Bert is particularly effective in capturing complex text sentiment [26,27]. These hybrid models utilize BERT’s deep semantic understanding ability and LSTM/GRU’s sequence processing ability to significantly improve sentiment classification accuracy.

The application of BERT in the academic world is also remarkable. It can understand complex academic language and terminology through pre-trained models and effectively identify key information such as the quality of academic papers, research innovation, and rigor of methods, which provides important support for editors and researchers in reviewing and screening academic papers.

## 4. Methodology

**Figure 3:**
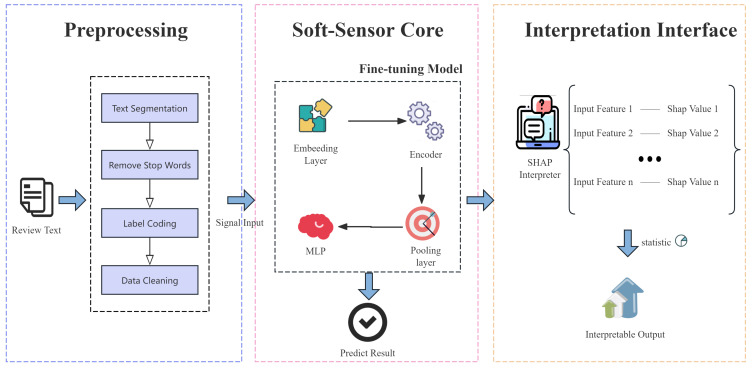
The overall framework of proposed interpretable soft-sensor for dissertation peer review.

This section introduces the datasets and models used. After that, we describe the approach used to analyze word significance. Figure 3 presents the overall architecture of the soft-sensor framework.

### 4.1. Original Signal Data

The research object of this study consists of a dataset comprising 15,923 master’s thesis evaluation records from the past five years at a university. This dataset covers 28 faculties and 171 disciplines.

Each evaluation record adopted in this dataset originated from manual annotations by experts in the field, forming a rich and multidimensional comprehensive assessment. The data structure includes both quantitative scores and categorical grades. Specifically, these were graded on a categorical scale (A–D) for their overall quality, with supporting scores across several critical dimensions: topic selection, foundational theory and specialized knowledge, scientific research capability and technological innovation, and thesis standardization. Crucially, the dataset also contains unstructured textual feedback from experts, comprising both academic comments that summarize strengths and sections on shortcomings and suggestions for improvement.

**Figure 4:**
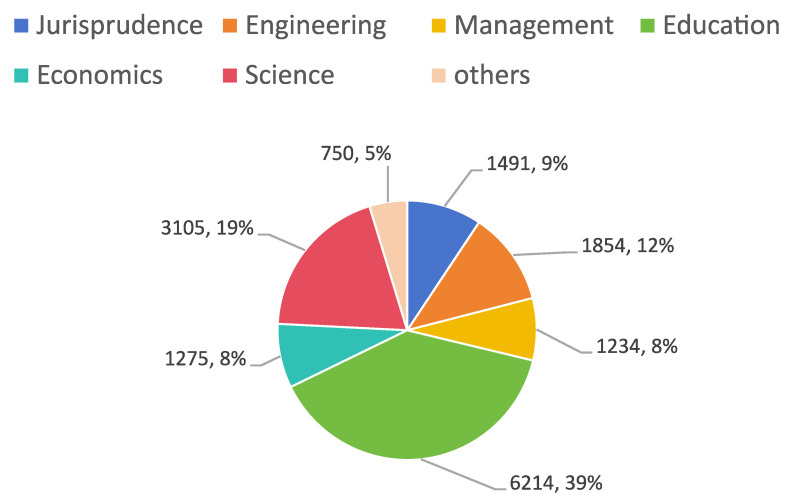
The proportion of each subject in the dataset.

To facilitate subsequent analysis, this study categorized the relevant data into six main categories: engineering, management, economics, education, jurisprudence, and science, as well as a few additional subject categories. The proportion of subjects is as shown in Figure 4. Each evaluation record is labeled with categorical grades A–D. The evaluations were conducted by experts who assessed various aspects of the theses, including topic selection, innovation, theoretical and specialized knowledge, as well as scientific research and writing proficiency. The data is authentic and comprehensive, providing a robust foundation for analysis. This corpus of review texts serves as the raw input signal for our academic soft-sensor.

### 4.2. Signal Preprocessing

**Figure 5:**
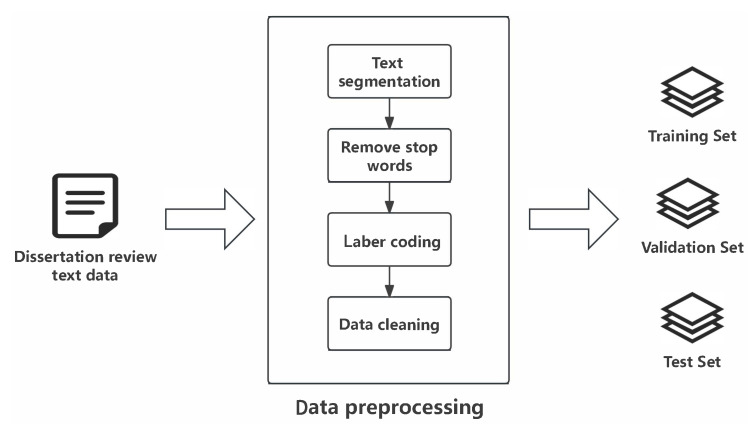
Preprocessing of thesis text data.

To ensure the quality and consistency of the input signal fed into the sensing core, the raw text data undergo a series of preprocessing and standardization procedures. These steps are designed to facilitate the construction of a BERT-based model for academic paper review, with the overall preprocessing pipeline illustrated in Figure 5. Initially, text tokenization is performed using the jieba segmentation tool to decompose the text into discrete words. Subsequently, non-informative stopwords are removed based on the comprehensive Baidu stopwords list, effectively eliminating high-frequency yet low-information terms. This process is crucial for reducing textual noise and highlighting the prominence of meaningful features, thereby significantly enhancing the model’s ability to comprehend and predict textual content with greater accuracy.

Furthermore, label encoding is applied to convert the original paper grades (‘A’, ‘B’, ‘C’, ‘D’) into binary classification labels. Specifically, grades ‘A’ and ‘B’ are assigned the class 1, while ‘C’ and ‘D’ are assigned the class 0. This mapping simplifies the training and prediction procedures and facilitates a more interpretable output from the classification model.

To ensure the quality of data, we undertake data cleaning measures. Excessively long paragraphs are truncated or segmented to adhere to the 512-token limit of the BERT model. This approach prevents errors or performance degradation caused by excessively lengthy text inputs, thereby maintaining the stability and efficiency. This process results in a clean, standardized textual signal that is suitable for ingestion by the BERT model.

Finally, the dataset is divided into training, validation, and test sets in the ratios of 70%, 15%, and 15%, respectively.

### 4.3. The Sensing Core: Feature Encoding and Inference with an Enhanced BERT Model

The core of the proposed soft-sensor is a modified BERT model, which serves as a powerful feature extractor and inference engine. It maps preprocessed textual tokens into contextually enriched representations, from which the final evaluation outcome is derived.

**Figure 6:**
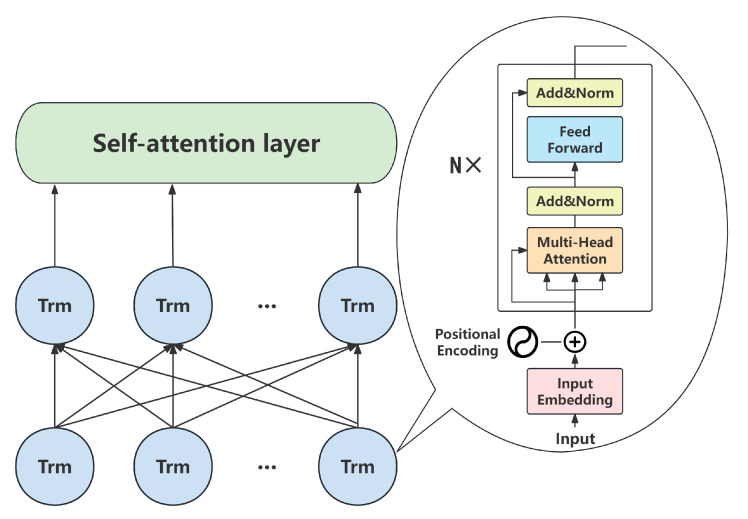
BERT model with additional self-attention layer.

As illustrated in Figure 6, an additional global self-attention layer is incorporated on top of the BERT encoder to function as a signal refinement module. This addition enables the architecture to perform a second, more focused integration of the encoded features, thereby enhancing its ability to capture long-range dependencies and key phrases in the review text—a critical capability for a sensor that discerns nuanced academic qualities.

During the training stage, the model was optimized using the AdamW optimizer with an initial learning rate of 5 × $10^{-5}$. The optimizer was configured with beta parameters (0.9, 0.999), an epsilon value of 1 × $10^{-8}$, and a weight decay of 0.01. Gradient clipping was applied with a maximum norm of 1.0 to stabilize the training process. The training ran for 15 epochs, using BCELoss as the loss function with a batch size of 32. A random seed of 42 was applied to ensure reproducibility, and the BERT layers were set with freeze_bert = False to allow full fine-tuning. Under this configuration, a pooling layer extracts a global feature representation of the input sequence. A special classification token ([CLS]) is inserted at the beginning of the sequence. After being processed through the Transformer encoder and the global self-attention layer, the output feature vector corresponding to this token is aggregated into a holistic representation of the entire sequence. This representation encapsulates the overall semantic information of the text and serves as a pivotal input for downstream classification tasks.

Subsequently, the refined feature representation from the [CLS] token is fed into a Multi-layer Perceptron (MLP) that acts as the final inference unit, mapping the high-dimensional features to a binary prediction. The MLP comprises multiple fully connected layers, typically including one or more hidden layers. Between these layers, a dropout layer is applied to mitigate overfitting by randomly turning off a subset of neurons during training, thereby improving generalization. The output layer consists of a linear projection followed by a Sigmoid activation function, which produces a probability score between 0 and 1 indicating the likelihood of the input text belonging to the positive class.

Leveraging the robust feature extraction capabilities of the BERT model, combined with nonlinear transformation via the MLP, the proposed soft-sensor demonstrates significantly enhanced performance in binary classification tasks. This architecture allows the sensor to not only encode complex textual patterns but also perform reliable inference on nuanced academic features.

This improvement in predictive performance substantiates more trustworthy SHAP-based interpretations of feature importance, reduces model-induced uncertainty, and clarifies associations between input features and outcomes. As a result, the soft-sensor can more accurately identify and articulate critical phrases and contextual cues that drive decisions, strengthening its role as an interpretable and effective tool for academic quality assessment.

### 4.4. The Interpretation Interface: Explaining Predictions with SHAP

**Figure 7:**
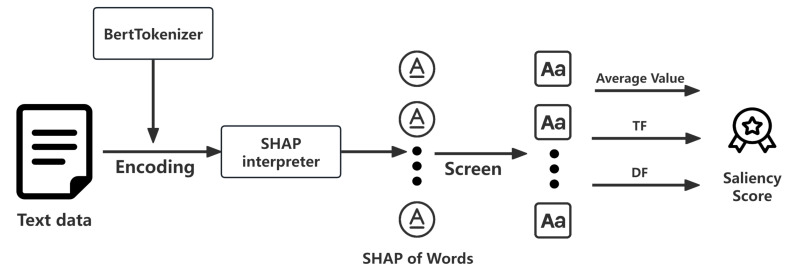
Flow chart of SHAP prediction explaining.

To make the predictions of the sensing core interpretable and uncover the latent evaluation dimensions, we employ SHAP as the calibration and explanation interface of our soft-sensor. This interface decomposes the BERT model’s output to quantify the contribution of each input feature (word), effectively reverse-engineering the black-box inference process. An overview of the process is shown in Figure 7.

A SHAP interpreter is instantiated to evaluate the sensing core’s response to individual input tokens. The SHAP values are computed through a transformation process wherein textual data are encoded via a tokenizer to ensure compatibility with the model architecture. By integrating the encoding process and tokenizer, the interpreter systematically quantifies the influence of each feature on the overall result. The resulting SHAP values assign a quantitative measure to each word’s contribution to the final prediction, thereby clarifying the model’s decision-making process and facilitating improved interpretation.

During the data screening and statistical processing phase, non-letter characters, stop words, non-nouns, and other lexically irrelevant elements were excluded to ensure data accuracy and relevance. The occurrence frequency of each word, along with the cumulative SHAP value associated with it, was computed to form the basis of saliency analysis. Additionally, the frequency of each word across documents and its document frequency were assessed to evaluate the term’s distribution within the corpus and its importance in the decision process.

Based on these metrics, a composite saliency score was calculated for each term by integrating its average SHAP value, term frequency (TF), and document frequency (DF) through a mathematically rigorous formulation. The composite saliency score for each term *w* is defined as

$$\mathrm{SaliencyScore}\left(w\right)=\left(\frac{\sum_{i=1}^{N}\left|{\mathrm{SHAP}}_{i}\left(w\right)\right|}{\mathrm{TF}(w)}\right)\times \;\left(\frac{\mathrm{DF}(w)}{\max_{v\in V}\mathrm{DF}\left(v\right)}\right)$$

The former component represents the mean absolute SHAP value per occurrence of term *w*, calculated by dividing the cumulative absolute SHAP values by TF. This normalization ensures that terms with high frequency but low per-occurrence impact are appropriately weighted, preventing the overemphasis of commonly occurring but individually insignificant terms. The latter component quantifies the prevalence of term *w* across the document corpus by dividing its DF by the maximum document frequency observed across all terms in the corpus. This ratio, bounded between 0 and 1, emphasizes terms that exhibit broad contextual relevance rather than those significant only within limited textual contexts.

Following this computation, a final normalization step is applied where all scores are scaled to the interval [0, 1] by dividing each score by the maximum observed value. The resulting scores were then sorted in descending order of significance. This comprehensive analytical process enables the systematic filtering and ranking of salient keywords based on their aggregated SHAP values, providing a human-interpretable output that reveals the key linguistic signals relied on for its assessment while ensuring statistical robustness and contextual relevance across the document corpus.

Furthermore, the SHAP-based keyword extraction process is entirely automated, requiring no manual intervention. To validate the distinctiveness of the selected keywords, we compared them with those generated by traditional methods such as TF-IDF. The results indicate that SHAP effectively captures contextually salient terms that correspond more closely with expert assessments, while TF-IDF tends to emphasize frequently occurring yet less distinctive words. This comparison confirms that our approach yields more semantically meaningful and review-specific insights.

## 5. Evaluation

### 5.1. Evaluation Metric

This study quantitatively evaluates the classification accuracy and reliability of the BERT model. For text classification tasks, standard performance metrics are employed, including accuracy (Acc), precision (Prec), recall (Rec), and F1-score (F1). Acc denotes the proportion of correctly classified samples out of the total. Prec represents the ratio of true positive predictions among all samples predicted as positive. Rec measures the fraction of actual positive samples that are correctly identified. The F1-score is the harmonic mean of precision and recall, providing a balanced assessment of the model’s performance. These metrics collectively evaluate the inference accuracy of the soft-sensor, ensuring reliable quantification of target variables from textual inputs. The calculation formulas for each metric are as follows:

(1) $$\mathrm{Precision}=\frac{\mathrm{TP}}{\mathrm{TP}+\mathrm{FP}}$$

(2) $$\mathrm{Accuracy}=\frac{\mathrm{TP}+\mathrm{TN}}{\mathrm{TP}+\mathrm{TN}+\mathrm{FP}+\mathrm{FN}}$$

(3) $$F1=2\times \frac{\mathrm{Precision}\times \mathrm{Recall}}{\mathrm{Precision}+\mathrm{Recall}}$$

(4) $$\mathrm{Recall}=\frac{\mathrm{TP}}{\mathrm{TP}+\mathrm{FN}}$$

where TP and TN represent the number of true positive and true negative samples, while FP and FN represent the number of false positive and false negative samples, respectively.

**Figure 8:**
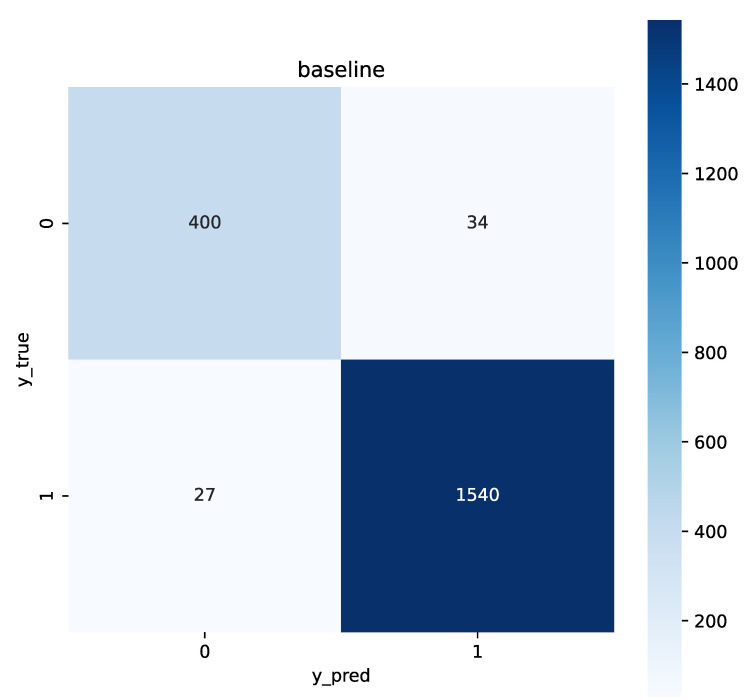
Confusion matrix for the binary classification model.

**Figure 9:**
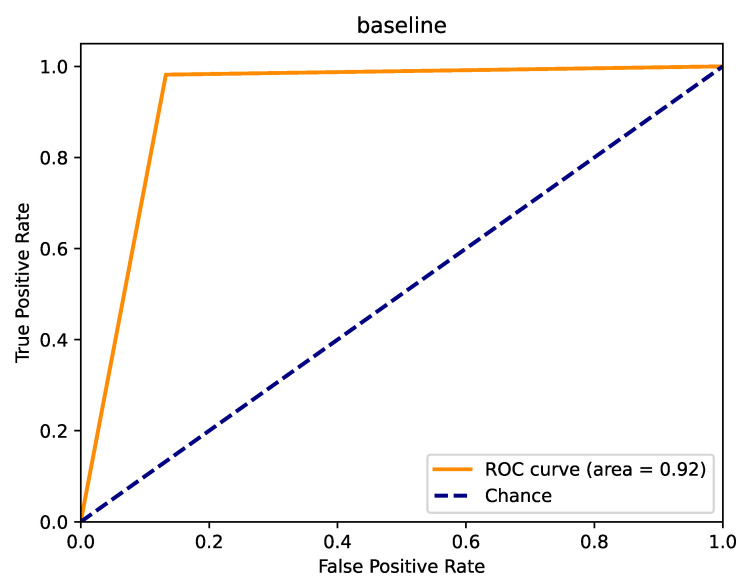
Receiver Operating Characteristic (ROC) curve.

Our experiments rigorously evaluate the model’s performance on binary classification tasks, focusing on key metrics such as accuracy, precision, recall, and F1-score. To provide a deeper insight into the classification abilities, we integrated a confusion matrix (Figure 8) and a Receiver Operating Characteristic (ROC) curve (Figure 9) into our analysis. The confusion matrix offers a detailed view of the model’s prediction accuracy across different classes, highlighting true positives, false positives, true negatives, and false negatives. This matrix effectively illustrates the balance between sensitivity and specificity achieved by our model.

Furthermore, the ROC curve analysis complements this by depicting the trade-off between the true positive rate (sensitivity) and the false positive rate (1-specificity) at various threshold settings. The area under the ROC curve (AUC) provides a single scalar value that summarizes the overall performance across all classification thresholds, indicating excellent discriminatory power.

These results substantiate the effectiveness of our proposed method and lay a robust foundation for further research and analysis. The comprehensive evaluation through both the confusion matrix and ROC curve not only confirms the high classification accuracy of our model but also demonstrates its reliability and robustness in handling binary classification tasks.

### 5.2. Performance Comparison

In this study, the modified BERT model was adopted for peer-review text classification. To evaluate the performance of our model, we compared it with several state-of-the-art text classification models, including support vector machines (SVM), BERT, RoBERTa, XLNet, Small-Text [28], TextRCNN, and two fine-tuning large language models: LLaMa3.1-8B and Qwen2.5-7B. Under identical hardware and software configurations, all models were trained and evaluated on the same dataset split, following consistent data preprocessing steps to guarantee the comparability of the results. The experimental environment is shown in Table 1.

The superior performance of the modified BERT model, as shown in Table 2, validates the effectiveness of our proposed architecture as a reliable sensing core. It should be noted that the SVM benchmark model has a slightly higher recall rate than the modified model in this study, which reflects the trade-off between precision and recall in the model. Our model sacrifices a small amount of recall rate in exchange for significant improvements in precision, accuracy, and F1-score.

To further evaluate the generalizability of our proposed method, we identified several top-performing models from the table above and evaluated them on the PeerRead dataset [29]. The results presented in Table 3 confirm that the advantage of our model is not limited to a single benchmark.

### 5.3. Result Analysis

The soft-sensor framework automatically extracts high-saliency keywords, which represent the most influential signals for inferring latent academic quality. To explore disciplinary distinctions in the focus of the review, we classified the data into six major categories and identified their corresponding keywords along with significance scores. As shown in Table 4, the top three keywords and their scores in each discipline reflect the primary input signals used by the sensor to assess academic quality. Additional details for each discipline are provided in the Appendix A. Furthermore, we performed a manual analysis of these salient keywords to connect computational output with substantive academic evaluation. This approach revealed different thematic priorities across the six disciplines, offering practical insight to improve dissertation quality. The detailed analysis is presented below.

1. **Engineering:** A high-quality engineering dissertation begins with an innovative topic rooted in practical needs, develops through systematic and rigorous research validation, and culminates in a coherent narrative that highlights its application value. The evaluation of engineering dissertations emphasizes research innovation—whether demonstrated through novel technical solutions or improvements to existing technologies—reflecting the discipline’s commitment to driving progress. Significant effort invested in the research should also be objectively documented. The technical content must be thorough and precise, demonstrating mastery of core technologies, key processes, and practical skills. Practical relevance is essential, with a focus on addressing real-world engineering challenges and delivering applicable value. Finally, feasibility, economic viability, safety, reliability, and other critical metrics must be rigorously evaluated through well-defined methods and procedures. A successful engineering dissertation effectively integrates these elements, particularly by combining innovation with practical implementation details and a comprehensive evaluation framework.
2. **Management:** A high-quality management dissertation must be grounded in real-world managerial practices. Through in-depth dialog with existing theories and the application of rigorous scientific methods, it is expected to yield profound insights that demonstrate both theoretical innovation and practical relevance. The evaluation of management dissertations places a strong emphasis on practical applicability and real-world value. While a solid theoretical foundation is essential, its true worth lies in guiding actionable strategies—such as transforming conceptual models into decision-making tools. The research methodology must be rigorous and appropriately selected, with scientific data analysis reflecting the empirical nature of management studies. Validating theories through carefully chosen case studies is critical, and these require thorough analysis to ensure relevance. This unique perspective should provide actionable insights, such as benchmarking frameworks or change initiatives, that directly enhance professional practice.
3. **Economics:** A high-quality economics dissertation begins with a well-crafted research question that integrates both practical relevance and theoretical significance. It develops through a rigorous research design capable of effectively identifying causal relationships and culminates in a profound interpretation and discussion of the economic implications underlying the empirical findings. The evaluation of economics dissertations places strong emphasis on adherence to disciplinary norms, particularly in data processing and quantitative analysis. Additionally, economics highly values logical reasoning throughout the paper. The research topic should demonstrate innovation, and theoretical analysis must be both in-depth and original, contributing to the advancement of existing theories. Data quality is also critical; it is important to ensure not only the representativeness and reliability of the data, but also the standardization of data processing and analytical procedures. Finally, the literature review section plays a vital role in an economics dissertation. It requires a comprehensive understanding of the current state of the discipline. It offers unique insights into relevant scholarly works, thereby connecting past research with future directions.
4. **Education:** A high-quality educational dissertation must be grounded in authentic educational contexts. Through profound engagement with theoretical frameworks and the application of rigorous and ethically sound methodologies, it should ultimately yield outcomes that embody both theoretical insight and practical wisdom, thereby genuinely contributing to human development and educational progress. The evaluation of educational dissertations places particular emphasis on structural integrity and coherence, with content expected to be presented in an organized and layered manner. Additionally, adherence to academic conventions—such as proper citation and formatting—is essential. The dissertation should demonstrate rigorous theoretical reasoning and persuasive argumentation, reflecting the importance placed on cultivating students’ critical thinking skills and capacity for clear expression. Research methods must be scientific and appropriately chosen, with an emphasis on accuracy in data analysis. Ultimately, the content should offer a comprehensive and in-depth examination of a specific issue, while also encompassing key domains within the discipline.
5. **Jurisprudence:** A high-quality juristic dissertation begins with a precise and contentious research question, develops through a logically rigorous and systematically layered argumentation guided by a central thesis, and culminates in a clear contribution to academic knowledge or the advancement of legal practice. The evaluation of a legal dissertation places great emphasis on strict adherence to scholarly standards and writing conventions, reflecting the discipline’s commitment to precision and clarity. The chosen topic must be both relevant and capable of advancing legal theory or practice. Depth of theoretical inquiry is essential, and the content must be structured with rigorous logic to enhance coherence and persuasiveness. Furthermore, the research’s practical and theoretical value should be clearly demonstrated, supported by well-selected references that lend authority to the discussion within the broader context of legal scholarship. These stringent requirements underscore the discipline’s dedication to cultivating meticulous and forward-looking legal scholarship.
6. **Science:** A high-quality scientific dissertation originates from a cutting-edge and well-defined scientific question, develops through exceptionally rigorous, transparent, and replicable research methodologies, and culminates in a profound interpretation of the underlying scientific mechanisms of the findings, clearly defining its significant contribution to expanding the boundaries of human knowledge. In reviewing scientific papers, great emphasis is placed on the scientific integrity of experimental data. The acquisition, processing, analysis, and interpretation of data must adhere to stringent standards, reflecting the principles of scientific empiricism and evidence-based conclusion-making. Experimental methods must be scientifically sound and meticulously designed, with standardized implementation. The importance of theoretical analysis should not be overlooked; it must demonstrate both depth and originality, enabling meaningful breakthroughs within existing theoretical frameworks. Furthermore, science emphasizes the use of mathematical models and other tools to uncover intrinsic laws, employing concise theories to explain complex phenomena—a reflection of the discipline’s pursuit of fundamental principles. Through astute topic selection, in-depth content analysis, and rigorous referencing, each component of the research process contributes to shaping work that is both informative and transformative, thereby driving continuous progress in scientific inquiry.

Across all analyzed disciplines, common keywords like “standard,” “topic selection,” and “theory” frequently emerge with high saliency scores, showcasing universal scholarly values such as rigor, relevance, and theoretical grounding. However, each discipline also displays unique prioritizations reflective of its specific scholarly and practical concerns. This analysis not only aids in understanding the core focus areas within each field but also assists in aligning future research endeavors with these expert-identified priorities.

## 6. Discussion

In the current era of rapid development of artificial intelligence technologies, represented by LLMs, this study proposes an interpretable soft-sensor framework based on BERT and SHAP. The core objective of this framework is to provide a stable, interpretable, and auditable discriminative foundation for academic evaluation. Unlike directly relying on generative models for end-to-end assessment, this framework aims to construct a reliable and transparent evaluation core by integrating semantic encoding and attribution analysis deeply. This core can serve as a crucial quality calibration module in future intelligent evaluation systems, used to verify, assist, and even constrain the “hallucinations” and inconsistencies that may exist in generative models. Thus, it can establish its unique value in the academic evaluation ecosystem driven by artificial intelligence.

However, although this framework is designed to achieve reliable perception and interpretable reasoning, its practical application still has several limitations. Firstly, the dataset used in this study mainly comes from a single institution. Although it encompasses multiple disciplines, the uneven distribution of disciplines and the differences in evaluation criteria among them may, to some extent, limit the model’s generalization ability. Additionally, the sample distribution of various categories in the dataset is unbalanced, which may result in the model’s recognition ability being weak in specific categories, making it difficult to achieve a more detailed and nuanced assessment of the papers’ quality.

In our future work, we will focus on expanding the evaluation dimensions of the model, moving from the current binary classification task to multi-class classification tasks, by modeling texts from more macro linguistic perspectives beyond the lexical level to provide more granular quality scores or level predictions. Secondly, we will actively explore the collaborative path with large language models. A promising direction is to combine the stable discrimination and attribution capabilities of this framework with the smooth generation and deep semantic understanding capabilities of generative models to build a hybrid intelligent system that not only has a reliable evaluation core but also can produce natural language comments, thereby promoting the academic review to a more scientific, transparent, and more instructive direction.

## 7. Conclusions

This study establishes an interpretable soft-sensor paradigm for academic assessment, conceptualizing BERT as a sensor core and SHAP as an explanatory interface. The proposed interpretable soft-sensor paradigm predicts dissertation evaluation results and quantifies the latent dimensions valued by experts, enabling a transparent and data-driven assessment process. It provides actionable insights for thesis quality improvement and supports the high-quality development of postgraduate education.

## Figures and Tables

**Table 1:** Experimental environment.

Hardware/Software	Specification
Operating System	Windows 10
GPU	NVIDIA GeForce RTX 4090
CPU	Intel(R) Core(TM)i7-11700K
Python	3.8
Pytorch	2.0.0 + CUDA 11.8
Encoding Format	UTF-8

**Table 2:** Comparison of the classification performance between the Modified BERT model and eight other models: SVM, BERT, RoBERTa, XLNet, Small-Text, TextRCNN, LLaMa3.1-8B, and Qwen2.5-7B.

Model	Acc (%)	Prec (%)	Recall (%)	F1 (%)
**Modified BERT**	**96.9515**	**97.8399**	**98.2770**	**98.0579**
SVM	90.6122	91.2942	98.6307	94.8207
BERT	88.3442	94.4918	90.6289	92.5201
RoBERTa	89.4947	92.7590	94.0506	93.4003
XLNet	89.2446	93.6528	92.5143	93.0801
Small-Text	91.3631	93.3172	97.0472	95.1456
TextRCNN	91.1865	94.5018	95.3684	94.9304
LLaMa3.1-8B	95.8890	97.2896	98.4127	97.8479
Qwen2.5-7B	95.8076	97.3128	98.2990	97.8034

**Table 3:** Comparison of the classification performance in the PeerRead dataset.

Model	Acc (%)	Prec (%)	Recall (%)	F1 (%)
**Modified BERT**	**92.0074**	**92.1659**	**88.4956**	**90.2935**
BERT	81.2268	76.2411	86.3454	80.9793
Small-Text	90.5812	89.3939	87.1921	88.2793
TextRCNN	71.4640	71.0999	71.0757	71.0873
LLaMa3.1-8B	76.2195	74.8837	71.8750	73.3485

**Table 4:** Selected keywords and scores for each discipline (for full details, see Appendix A).

Discipline	Keyword 1	Score 1	Keyword 2	Score 2	Keyword 3	Score 3
Engineering	standard	1.0	topic selection	0.857	originality	0.740
Management	topic selection	1.0	theory	0.820	content	0.628
Economics	standard	1.0	logic	0.667	topic selection	0.642
Education	structure	1.0	standard	0.860	method	0.830
Jurisprudence	standard	1.0	topic selection	0.833	theory	0.733
Science	standard	1.0	topic selection	0.843	data	0.601

## Data Availability

The data presented in this study are available on request from the corresponding author due to privacy.

## Appendix A. Detailed Keyword for Each Discipline

**Table A1:** Keywords and saliency scores for Engineering.

Keyword	Saliency Score	Keyword	Saliency Score
standard	1.0	method	0.593
topic selection	0.857	value	0.540
originality	0.740	structure	0.477
work load	0.708	performance	0.450
content	0.612	theory	0.448

**Table A2:** Keywords and saliency scores for Management.

Keyword	Saliency Score	Keyword	Saliency Score
topic selection	1.0	logic	0.502
theory	0.820	structure	0.496
content	0.628	foundation	0.493
method	0.624	status quo	0.389
standard	0.590	countermeasure	0.321

**Table A3:** Keywords and saliency scores for Economics.

Keyword	Saliency Score	Keyword	Saliency Score
standard	1.0	structure	0.509
logic	0.667	originality	0.459
topic selection	0.642	practical value	0.408
theory	0.622	foundation	0.388
reference	0.558	conclusion	0.342

**Table A4:** Keywords and saliency scores for Education.

Keyword	Saliency Score	Keyword	Saliency Score
structure	1.0	topic selection	0.609
standard	0.860	content	0.477
method	0.830	theory	0.403
logic	0.823	reference	0.377
thought	0.649	value	0.346

**Table A5:** Keywords and saliency scores for Jurisprudence.

Keyword	Saliency Score	Keyword	Saliency Score
standard	1.0	logic	0.609
topic selection	0.833	value	0.589
theory	0.733	argument	0.584
content	0.725	view	0.515
structure	0.642	reference	0.460

**Table A6:** Keywords and saliency scores for Science.

Keyword	Saliency Score	Keyword	Saliency Score
standard	1.0	theory	0.513
topic selection	0.843	significance	0.503
data	0.601	workload	0.502
content	0.663	value	0.462
structure	0.580	reference	0.461
